# Effects of pre-surgery physiotherapy on walking ability and lower extremity strength in patients with degenerative lumbar spine disorder: Secondary outcomes of the PREPARE randomised controlled trial

**DOI:** 10.1186/s12891-019-2850-3

**Published:** 2019-10-24

**Authors:** Maria Fors, Paul Enthoven, Allan Abbott, Birgitta Öberg

**Affiliations:** 1 Department of Medical and Health Sciences, Division of Physiotherapy, Faculty of Medicine and Health Sciences, Linköping University, SE-581 83 Linköping, Sweden; 2 Department of Activity and Health, and Department of Medical and Health Sciences, Linköping University, Linköping, Sweden

**Keywords:** Degenerative lumbar spine disorder, Low Back pain, Physiotherapy, Rehabilitation, Exercise, Walking ability, Strength

## Abstract

**Background:**

Degenerative lumbar spine disorders are common among musculoskeletal disorders. When disabling pain and radiculopathy persists after adequate course of rehabilitation and imaging confirms compressive pathology, surgical decompression is indicated. Prehabilitation aiming to augment functional capacity pre-surgery may improve physical function and activity levels pre and post-surgery. This study aims to evaluate the effect and dose-response of pre-surgery physiotherapy on quadriceps femoris strength and walking ability in patients with degenerative lumbar spine disorders compared to waiting-list controls and their association with postoperative physical activity level.

**Method:**

In this single blinded, 2-arm randomised controlled trial, 197 patients were consecutively recruited. Inclusion criteria were: MRI confirmed diagnosis and scheduled for surgery due to disc herniation, lumbar spinal stenosis, degenerative disc disease or spondylolisthesis, ages 25-80 years. Patients were randomised to 9 weeks of pre-surgery physiotherapy or to waiting-list. Patient reported physical activity level, walking ability according to Oswestry Disability Index item 4, walking distance according to the SWESPINE national register and physical outcome measures including the timed ten-meter walk test, maximum voluntary isometric quadriceps femoris muscle strength, patient-rated were collected at baseline and follow-up. Parametric or non-parametric within and between group comparisons as well as multivariate regression was performed.

**Results:**

Patients who received pre-surgery physiotherapy significantly improved in all variables from baseline to follow-up (*p* < 0.001 – *p* < 0.05) and in comparison to waiting-list controls (p < 0.001 – *p* < 0.028). Patients adhering to ≥12 treatment sessions significantly improved in all variables (p < 0.001 – *p* < 0.032) and those receiving 0-11 treatment session in only normal walking speed (p0.035) but there were no significant differences when comparing dosages. Physical outcome measures after pre-surgery physiotherapy together significantly explain 27.5% of the variation in physical activity level 1 year after surgery with pre-surgery physical activity level having a significant multivariate association.

**Conclusion:**

Pre-surgery physiotherapy increased walking ability and lower extremity strength in patients with degenerative lumbar spine disorders compared to waiting-list controls. A clear treatment dose-response response relationship was not found. These results implicate that pre-surgery physiotherapy can influence functional capacity before surgical treatment and has moderate associations with maintained postoperative physical activity levels mostly explained by physical activity level pre-surgery.

**Trial registration:**

NCT02454400. Trial registration date: August 31st 2015, retrospectively registered.

## Background

Low back pain is a common musculoskeletal disorder that has become a public health problem. The lifetime prevalence is estimated to 39% [1]. Although only 10% of low back pain disorders are diagnosed as lumbar spinal stenosis (LSS) or disc herniation, they are the most common cause for spinal surgery, followed by degenerative disc disease (DDD) and spondylolisthesis [2, 3]. In the Swedish Spine register of spinal surgery (SWESPINE), the diagnosis LSS, disc herniation, spondylolisthesis and DDD are termed as degenerative lumbar spine disorders [3].

Degenerative lumbar spine disorders may cause pain and sensorimotor deficits in the lower extremities, affecting physical and self-rated extremity function [4, 5]. Impaired walking ability with regards to distance, speed and pattern is often seen in these patients [6–9]. Symptom-related activity restriction may also occur with implications for overall health and increased risk of comorbidities related to inactivity [9, 10]. Limited walking ability is one of the main reasons of patients with LSS to seek care [5, 10, 11] and a progressive reduction of walking distance, often due to leg pain, is according to some studies an indication for back surgery [12, 13].

Spinal surgery as treatment for degenerative lumbar spine disorders has over the last decade increased excessively in Sweden and the rest of the world [3, 14]. Only 10% of the studies on the effect of surgery report about non-surgical intervention prior to surgery for degenerative lumbar spine disorders [15]. However, it is standard practice in Sweden that an adequate period of primary care non-surgical treatment is trialed before decision making as recommended in the literature [16–20]. Prehabilitation on the other hand is aimed at augmenting functional capacity in patients selected for surgery and in that way possibly improve post-surgical outcomes [21, 22]. It is unknown how aspects of prehabilitation dosage may lead to these potential effects. In line with evidence-based guidelines for low back pain [23] and degenerative lumbar spine disorders [16–18], the effectiveness of structured physiotherapy in the prehabilitation context for surgical candidates has been investigated in the PREPARE trial [24, 25]. It was shown the pre-surgery physiotherapy decreased pain, risk of avoidance behavior and worsening of psychological well-being, and improved quality of life more than surgical waiting-list controls in the pre-surgical phase. Patient reported physical activity was significantly higher after pre-surgery and was maintained one year after surgery compared to the waiting-list controls [25].

Despite that one of the primary goals with treatments for degenerative lumbar spine disorders is to improve pain related disability, especially walking ability, only few studies investigating treatment for this patient group provide thorough evaluation with not only patient-reported outcomes but also objective physical assessments [9, 14, 26]. It is suggested that objective measures of function can complement self-rated measures to provide a comprehensive picture of patient disability [26]. However, a better understanding of how these measures are associated is required. In the PREPARE trial [24], objective physical assessments such as gait speed and lower extremity strength as well as patient-reported walking ability were collected as secondary outcomes during the prehabilitation phase conducted in the primary care setting. This to investigate if pre-surgery physiotherapy can augment pre-surgery physical capacity. Only self-reported measures could be performed post-surgery due to logistical reasons. The purpose of this report is therefore to investigate effectiveness of pre-surgery physiotherapy compared to waiting-list controls in the PREPARE trial [24] on walking ability and quadriceps femoris strength in patients with degenerative lumbar spine disorders following the intervention before surgery. An additional purpose is to investigate if differences exist between these physical outcomes based on the level of dosage adherence in prehabilitation and if pre-surgical physical measures have multivariate associations with self-reported physical activity level one year after surgery.

## Method

### Study design

This study is a single blinded, 2-arm, randomised controlled trial. The protocol has been published [24] and the patient reported core outcome set has been reported [25]. This paper presents secondary outcomes of muscle strength and walking ability. The CONSORT guidelines were followed for reporting [27]. The study was approved by the Regional Ethics committee (dnr 2012/167-31). Patients´ gave their written consent to participate in the study.

### Subjects and setting

Patients were consecutively recruited at the Spinal Clinic at the University Hospital in Linköping Sweden between October 2012 and March 2015. All patients who were referred to the Spine Clinic were examined by an orthopedic spine surgeon. Inclusion criteria were: age of 25-80 years, MRI confirmed diagnosis of disc herniation, LSS, DDD or spondylolisthesis (at least grade 4), scheduled for surgery, fluent in Swedish. Exclusion criteria were: indication for acute surgery, presence of severe spinal pathology, previous surgery on the same lumbar spinal level. A total of 242 patients met the inclusion criteria.

### Intervention

Patients in the waiting-list group received usual care which included information about the surgical procedure, postoperative rehabilitation and advice regarding continued physical activity. Patients in the physiotherapy group received the same usual care intervention and the pre-surgery physiotherapy one-hour session twice a week for 9 weeks containing:

1. Active physiotherapy according to a treatment-based classification (TBC) [28];
  - Specific exercise and mobilization, or b) Motor control exercises, or c) Traction. Treatment approach dependent upon assessment findings.
2. Tailor-made general exercise program performed in a gym supervised by a physiotherapist in one-on-one sessions. The program included strength-, cardiovascular- and mobility exercises. Dose and intensity of the exercise were set and progressed over time. The program was also individualized to the patients’ specific impairments.
3. Behavioral approach to increase activity level and decrease fear-avoidance behavior.
4. Daily physical activity for at least 30 min/day. The patient wrote a daily logbook over physical activity.

The interventions were performed at one of eleven physiotherapy public health care clinics in Östergötland County. The physiotherapists who delivered the intervention were trained and received directives for the treatment by two specialist physiotherapists. For each patient the physiotherapist followed a checklist with treatment and progression for each treatment-session. Modification of the treatment could be individually tailored. The intervention is further described in the study protocol by Lindbäck et al. [24].

### Outcome

Physical outcome measures, including patient-reported outcome measures and objective outcome measures, were collected at baseline and after 9 weeks intervention pre-surgery.

Walking ability was measured through gait speed and self-rated questions regarding walking ability. To assess walking speed a 5 -10 m distance is recommended in a wide range of populations [29, 30]. Gait speed in meters/second, was measured through a timed ten- meter walk test (10MWT). Patients walked 10 m on a straight path with 3 m for acceleration before and 3 m for deceleration after. Patients were asked to walk in their normal and fastest gait speed. Each pace was measured once. The 10MWT is reported to be a reliable and valid measurement for gait speed [31–33].

Self-rated walking ability was measured using item four of the Oswestry Disability Index (ODI) regarding how pain affects walking distance [34, 35]. There is evidence supporting its validity and reproducibility [36]. A question from SWESPINE, was used to evaluate self-rated walking ability. The question was: “How far can you walk at normal walk speed?” There were four answer alternatives: 1. Less than 100 m. 2. 100-500 m. 3. 0.5 km – 1 km. 4. More than 1 km. Physical activity level was measured after pre-surgery intervention and one year after surgery by a question with 5 answer options ranging from very little physical activity to regular strenuous physical activity.

Strength in the lower extremities was tested in the quadriceps femoris muscle. Maximum voluntary isometric muscle force was measured with a dynamometer model Chatillon CSD 500 strength dynamometer (Ametek, Largo, FL, USA). The dynamometer is sensitive to small changes in muscle strength [37] with good reliability [37–39]. The same measurement procedure was used as in former studies measuring normative values of maximum voluntary isometric force using a dynamometer [40, 41]. The test was repeated two times on the right and on the left leg. If the second test score was higher than the first, a third test was done. The highest peak torque obtained was recorded in kilogram (Kg).

### Randomisation

Block randomisation was used with sealed envelopes prepared for each randomisation block. An independent physiotherapist at the Spine Clinic performed the randomisation and informed the patient about group allocation.

### Blinding

The two physiotherapists who performed the measurements at baseline and follow up were blinded to the randomization. The patient and the treating physiotherapist could not be blinded for test condition.

### Statistical analysis

IBM SPSS® version 25 was used in performance of statistical analyses. Variables were tested for normality using Shapiro-Wilks test and skewness. For comparison of data at baseline and follow-up between physiotherapy- and waiting-list groups, Mann-Whitney U test was used for variables not normally distributed or categorical and Student’s t-test was used for continuous and normally distributed variables. For within group comparisons paired Student’s t-test or a Wilcoxon Signed-Rank test were used. A *P*-value of < 0.05 was considered as statistically significant. Cohen *d* effect size was calculated for change in group over time, where *d* ≥ 0.20 was considered a small, *d* ≥ 0.50 a medium and *d* ≥ 0.80 was a large effect size [42]. Hedges *g* was used to calculate effect size when the sample sizes where dissimilar [43]. Bivariate and multivariate associations between number of pre-surgery treatment sessions and physical outcome measures were analysed to investigate possible linear or non-linear associations with regards to dose-response curves. Stepwise linear regression analysis was used to evaluate the multivariate associations between pre-surgical physical outcome measures (independent variables = walking speed, Quadriceps strength, self-rated walking ability through ODI item 4 and a question from SWESPINE regarding walking distance) and physical activity level one year after surgery. In the first step, pre-surgery physical outcome measures were entered and as a second step pre-surgery physical activity level. The data assumptions required for linear regression modelling were test and confirmed.

Per protocol analyses was performed on data provided from patients’ adherent to at least 12 treatment sessions of the intervention. The trial’s a-priori sample size calculation was based on the ODI total score which was the primary outcome in the PREPARE Randomised controlled trial [24].

## Results

**Fig. 1:**
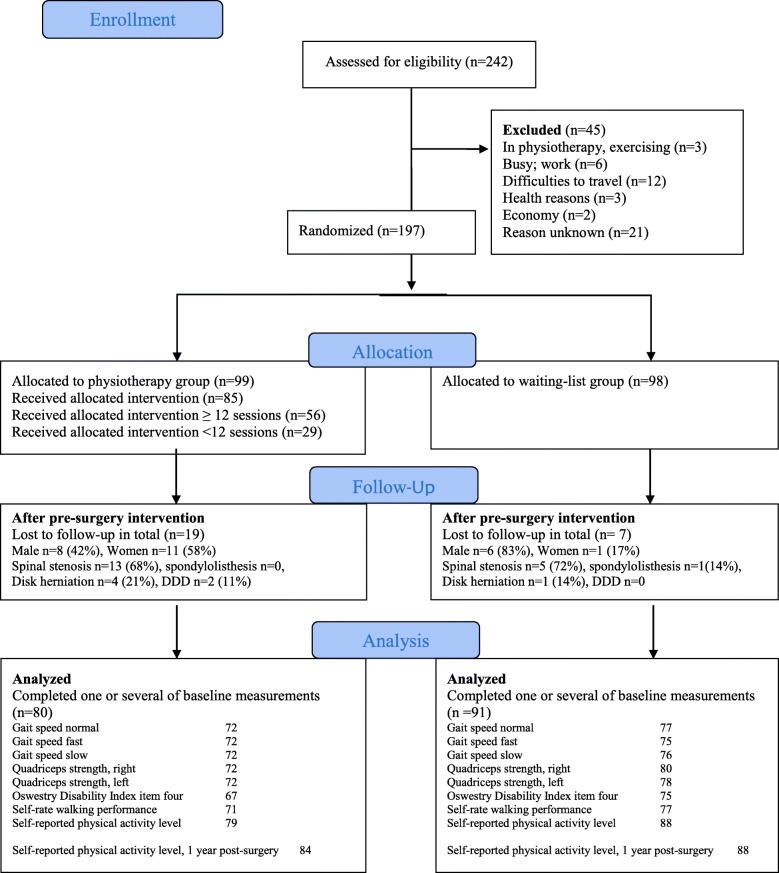
Consolidated Standards of Reporting Trials (CONSORT) flowchart diagram of the randomised controlled trial

One hundred ninety-seven patients with degenerative lumbar spine disorders were allocated to either waiting-list group (*n* = 98) or physiotherapy group (PT) (*n* = 99). A total of 171 patients were included in the per protocol analysis (Fig. 1).

No significant differences in the patients’ characteristics were found between the two groups at baseline (Table 1).

**Table 1:** Baseline characteristics for all recruited patients and patients who completed one or several outcome measures at follow-up. Presented in mean and standard deviation or frequency. Significance level for differences in between groups

	All recruited patients			Analysed patient		
	PT (*n* = 99)	Waiting-list (*n* = 98)	*P*-value	PT (*n* = 80*)	Waiting-list (*n* = 91*)	*P*-value
**Gender, men** (%)	45 (45)	47 (49)	0.725	37 (46)	45 (50)	0.573
**Age, years**	57.9 ± 13.3	61 ± 11.5	0.082	59.2 ± 12.5	60.9 ± 11.2	0.349
**Duration of current pain episode in months**	25.2 ± 2.7 (n = 98)	27.8 ± 3.9 (*n* = 97)	0.594	28.3 ± 3	29.3 ± 4.1	0.202
**Duration in months**	304.6 ± 29.7 (n = 80)	289.2 ± 29.3 (*n* = 87)	0.738	307.9 ± 29.6 (*n* = 64)	277.4 ± 28.6 (*n* = 81)	0.531
**Diagnosis** (%)						0.409
Spinal stenosis	59 (60)	70 (71)		52 (65)	66 (72)	
Disc herniation	23 (23)	17 (17)		13 (16)	15 (17)	
Spondylolisthesis	8 (8)	7 (7)		7 (9)	6 (7)	
DDD	9 (9)	4 (4)	0.286	8 (10)	4 (4)	
**Pain (Visual Analog Scale)**						
Back pain	55.4 ± 26.7 (*n* = 89)	58.7 ± 23.9 (*n* = 92)	0.514	54.5 ± 26.8 (*n* = 74)	58.2 ± 23.6 (*n* = 85)	0.363
Leg pain	65.4 ± 23.8 (*n* = 88)	64.8 ± 21.5 (n = 91)	0.620	63.8 ± 24.5 (n = 74)	63.8 ± 21.7 (*n* = 84)	0.983

Bold text ***p*** < .05

* Number of patients (n) in PT/Waiting-list group: Gait speed normal 72/77, Gait speed fast 72/75, Gait speed slow 72/76. Quadriceps Peak right 72/80, Quadriceps Peak left 72/78, ODI question four 67/75, Self-rate walking performance 71/77

The analyses revealed that the PT-group had a statistically significant improvement in gait speeds and in quadriceps femoris strength at follow-up directly after the 9 week pre-surgery intervention and in comparison to the waiting-list group. In the waiting-list group there were no significant changes in gait speeds between baseline and after 9 week pre-surgery intervention. There was a deterioration in maximum voluntary muscle force in both legs after intervention compared to baseline in the waiting-list group, with a right leg decrease in strength reaching statistical significance. There were no statistically significant differences between groups at baseline (Table 2).

**Table 2:** Means in gait speed (m/s) at normal and fast speed and in maximum voluntary muscle force (Kg) of the right and left m. Quadriceps at baseline and after 9 week pre-surgery intervention. Within- and between groups differences over time

		Baseline		Within group changes			Between group change	
	n	Mean (SD)	*p*-Value	Mean (SD) change from baseline	*p*-Value	Effect size*	Mean difference (95% CI)	*p*-Value
Gait speed, normal								
PT	72	1.10 (0.24)	0.112	- 0.09 (0.14)	**< 0.001**	- 0.34	0.07 (0.02 to 0.12)	**0.005**
Waiting-list	77	1.13 (0.24)		- 0.01 (0.17)	0.556	- 0.04		
Gait speed, fast								
PT	72	1.52 (0.44)	0.989	- 0.12 (0.22)	**0.001**	- 0.29	0.21 (0.07 to 0.26)	**< 0.001**
Waiting-list	75	1.53 (0.38)		0.05 (0.36)	0.222	0.12		
Quadriceps strength, right								
PT	72	22.20 (8.08)	0.958	- 1.74 (5.64)	**0.011**	- 0.11	2.80 (0.74 to 4.87)	**< 0.001**
Waiting-list	80	22.22 (9.70)		1.54 (6.51)	**0.026**	0.16		
Quadriceps strength, left								
PT	72	21.81 (8.62)	0.912	- 2.40 (7.31)	**0.006**	- 0.25	3.28 (1.30 to 5.20)	**0.003**
Waiting-list	78	21.80 (9.10)		0.38 (5.40)	0.368	0.01		

PT, physiotherapy group; SD, standard deviation; CI, confidence interval. Bold text *p* < **.05**. *Cohen *d*

The PT-group improved significantly in the ODI item four and the walking distance question from baseline to follow-up after pre-surgery intervention and in comparison to the waiting-list group (Table 3). There were no significant between group differences at baseline (ODI item four p 0.607, walking distance question p 0.558).

**Table 3:** Self-rated walking ability measured with Oswestry Disability Index (ODI) item four and self-rated walking distance at baseline and after 9 week pre-surgery intervention and between group changes. Changes within groups presented in n (%)

	PT			Waiting-list			Between group change
	Baseline	Follow-up	*p*-Value	Baseline	Follow-up	*p*-value	*p*-value
ODI item four	*n* = 67	n = 67		*n* = 75	n = 75		
Pain does not prevent walking	16 (24)	13 (20)		10 (13)	8 (10)		
Walking distance 1 km	19 (28)	32 (47)		28 (38)	27 (36)		
Walking distance 500 m	16 (24)	13 (20)		23 (31)	21 (28)		
Walking distance 100 m	15 (22)	9 (13)		10 (13)	11 (15)		
In need of crutches	1 (2)	0		4 (5)	8 (11)		
Mostly bedridden	0	0	**0.026**	0	0	0.139	**0.007**
Self-rated walking distance	*n* = 71	n = 71		*n* = 77	n = 77		
< 100 m	2 (3)	4 (5)	**0.046**	6 (8)	7 (9)	0.467	**0.028**
100 till 500 m	34 (48)	25 (35)		31 (40)	32 (41)		
0,5 - 1 km	12 (17)	11 (16)		22 (29)	22 (29)		
> 1 km	23 (32)	31 (44)		18 (23)	16 (21)		

Bold text *p* **< .05**

In a first step screening bivariate or multivariate associations (linear or non-linear) between number of pre-surgery treatment sessions and physical outcome measure showed no significant associations in regard to dose-response curves. Therefore, a further analysis investigating differences in a dichotomized variable for pre-surgery treatment sessions (0-11/≥12 sessions) was investigated. Patients who adhered to 12 or more treatment sessions showed statistically significant improvement in normal gait speed and fast gait speed and in maximum voluntary muscle force in the right and left Quadriceps. The patients who adhered to 0-11 treatment sessions had only a statistically significant improvement in normal gait speed. There were no statistically significant differences between groups at baseline and no differences in change over time between groups (Table 4).

**Table 4:** Means in the PT-group gait speed (m/s) at normal and fast speed and in maximum voluntary muscle force (Kg) of the right and left m. Quadriceps at baseline and after 9 week pre-surgery intervention divided in number of treatment sessions. Within- and between groups differences over time

	Patients	Baseline		Within group changes			Between group change		
	n, Treatment sessions (median, range)	Mean (SD)	*p*-Value	Mean (SD) change from baseline	*p*-Value	Effect size*	Mean difference (95% CI)	*p*-Value	Effect size** (95% CI)
Gait speed normal									
0-11 treatment sessions	17 (5, 0-10)	1.15 (0.14)	0.711	−0.06 (0.10)	**0.035**	- 0.37	0.04 (−0.04 to 0.12)	0.305	−0.312 (−0.635 - 0.012)
≥ 12 treatment sessions	55 (18, 12-23)	1.12 (0.27)		− 0.10 (0.15)	**< 0.001**	- 0.37			
Gait speed fast									
0-11 treatment sessions	17 (5, 0-10)	1.54 (0.32)	0.857	−0.05 (0.22)	0.299	- 0.21	0.08 (−0.04 to 0.19)	0.181	−0.372 (− 0.698 - -0.046)
≥ 12 treatment sessions	55 (18, 12-23)	1.52 (0.40)		−0.13 (0.21)	**< 0.001**	- 0.84			
Quadriceps strength, right									
0-11 treatment sessions	18 (5, 0-10)	25.23 (9.01)	0.069	−1.93 (7.50)	0.289	- 0.19	- 0.26 (−3.34 to 2.80)	0.865	0.014 (−0,277 – 0.36)
≥ 12 treatment sessions	54 (18, 12-23)	21.23 (7.58)		−1.67 (4.96)	**0.016**	- 0.20			
Quadriceps strength, left									
0-11 treatment sessions	18 (5, 0-10)	22.51 (8.30)	0.765	−3.82 (10.50)	0.141	- 0.36	- 1.90 (−5.80 to 2.10)	0.354	0.22 (−0.101 – 0.541)
≥ 12 treatment sessions	54 (18, 12-23)	21.80 (8.81)		−1.96 (5.96)	**0.019**	- 0.22			

*SD* standard deviation, *CI* confidence interval. Bold text p < **.05**. *Cohen *d,* **Hedges *g*

The patients who adhered to 12 or more treatment sessions had a statistically significant improvement in ODI item four and self-rated walking distance. The patients who adhered to 0-11 treatment sessions did not improve significantly (Table 5). There were no significant between group differences at baseline (ODI item four p 0.082, walking distance question p 0.346) or in change over time (ODI item four p 0.866, walking distance question p 0.542).

**Table 5:** Self-rated walking ability measured with Oswestry Disability Index (ODI) item four and self-rated walking distance at baseline and after 9 week pre-surgery intervention divided in number of treatment sessions. Changes within groups over time

	0-11 treatment session			≥ 12 treatment session		
	Baseline	Follow-up	*p*-Value	Baseline	Follow-up	*p*-Value
ODI item four						
Treatment sessions (median, range)	*n* = 18 (5, 0-10)	n = 18 (5, 0-10)	0.763	*n* = 49 (18,12-23)	n = 49 (18,12-23)	
Pain does not prevent walking	6 (33)	5 (28)		10 (20)	8 (16)	
Walking distance 1 km	7 (39)	8 (44)		12 (25)	24 (49)	
Walking distance 500 m	4 (22)	3 (17)		12 (25)	10 (21)	
Walking distance 100 m	1 (6)	2 (11)		14 (28)	7 (14)	
In need of crutches	0	0		1 (2)	0	
Mostly bedridden	0	0		0	0	**0.006**
Self-rated walking distance						
Treatment sessions (median, range)	*n* = 21 (5, 0-10)	n = 21 (5, 0-10)	0.783	*n* = 50 (18,12-23)	n = 50 (18,12-23)	
< 100 m	1 (5)	1 (5)		1 (2)	3 (6)	**0.032**
100 till 500 m	8 (38)	8 (38)		26 (52)	17 (34)	
0,5 - 1 km	3 (14)	2 (10)		9 (18)	9 (18)	
> 1 km	9 (43)	10 (47)		14 (28)	21 (42)	

Bold text *p* < .**05**

Pre-surgical gait speed, self-rated walking ability and Quadriceps strength together significantly explain 17.4% (*p* = 0.003) of variation in self-reported physical activity level one year after surgery in step 1. Adding pre-surgery physical activity level increased the explanatory value to 27.5% (p = < 0.001) in step 2. In step 1 no single physical outcome measure alone was significant but in step 2, pre-surgery physical activity level alone had a significant association with physical activity level one year post-surgery (β = 0.281 *p* < 0.001) (Table 6).

**Table 6:** Stepwise regression analysis estimating multivariate association between preoperative physical outcome measures and physical activity level (independent variables) and physical activity level one year after surgery (dependent variable) (*n* = 108)

	Step 1		Step 2	
Independent variable	Unstandardized Beta (SE)	*P*-value	Unstandardized Beta (SE)	*P*-value
Gait speed, normal	0.430 (0.57)	0.449	0.502 (0.53)	0.348
Gait speed, fast	0.203 (0.39)	0.604	−0.159 (0.38)	0.677
Quadriceps strength, right	0.021 (0.02)	0.325	0.020 (0.02)	0.330
Quadriceps strength, left	−0.011 (0.01)	0.550	−0.010 (0.01)	0.547
ODI item four	−0.123 (0.12)	0.309	−0.062 (0.11)	0.586
Self-rated walking distance	0.079 (0.13)	0.548	0.084 (0.12)	0.499
Physical activity level			0.281 (0.07)	**< 0.001**
	R Square = 0.174	**0.003**	R Square = 0.275	**< 0.001**

*SE* Standard Error, *ODI* Oswestry Disability Index. Bold text p < **.05**

## Discussion

Walking ability is an important outcome as it correlates with several function- and health outcomes [32, 44–46] and is reported to be predictive for spine surgical outcomes [7, 47, 48]. The use of objective and self-reported walking measures as secondary outcomes in the PREPARE study [24], allows to capture a broader view of walking ability.

The result of the present study showed a significant improvement in gait speed, self-rated walking ability and in maximum voluntary isometric force in quadriceps femoris after 9 weeks pre-surgery physiotherapy interventions compared to waiting-list controls prior to surgery. The effect sizes were small but the effects might still be of importance in this group of patients with a quite high mean age. According to previous literature a change in gait speed of 0.10-0.17 m/s is said to be a minimal clinically important difference across multiple patient groups [49]. The improvement in normal gait speed for the PT-group was similar to these results while change in fast gait speed surpassed this level both for within group and between group change. Thus, the study results indicate that patients with degenerative lumbar spine disorders with large physical impairment and who are candidates for surgical treatment still have the potential to improve their physical function and benefit from physiotherapy. This suggests that there is a potential to improve physical function before surgery. The secondary analysis of the PREPARE study supports the previously reported effects on self-reported outcomes [25].

The current study is one of few studies including physical function outcomes with both self-rated and objective measurements. The results are in line with previous studies evaluating physical function in these patient groups in a prehabilitation phase [25, 50]. Only Nielsen et al. [50] have evaluated prehabiliation intervention in patients with degenerative lumbar spine disorders with physical performance functional tests. Unlike the results in the present study, Nielsen et al. [50] reported that only the self-rated physical function improved after pre-surgery intervention.

Numerous physiotherapy interventions have been recommended for patients with degenerative lumbar spine disorders [14, 17, 19, 20, 51, 52]. The physiotherapeutic intervention tested in the present study is multi-dimensional and targets several aspects of the biopsychosocial model [53]. The intervention is based on a treatment-based classification, the exercise is tailor-made and with a behavioral approach to meet the different demands in a heterogenous population. This does not allow to draw conclusions about single interventions. When considering a dose-response relationship, participation in > 12 treatment sessions resulted in statistically significant improvements but not statistically significant compared with < 11 treatment sessions concerning physical measures. When considering effect sizes, the larger change in fast gait speed after > 12 treatment sessions and the interpretation of between group effect size confidence intervals = − 0.372 (95%CI -0.698 - -0.046) may support a potential for statistically significant difference between groups if a larger sample size was included in the study. The reasoning for analysing a cut-off of 12 treatment session can be supported by a previously published meta-regression analysis showing improvement of pain and function after similar interventions for degenerative musculoskeletal conditions such as osteoarthritis [54].

Previous results from the PREPARE study published by Lindbäck et al. [25] show an improvement in physical activity level and in fear-avoidance beliefs after the pre-surgery intervention. Fear avoidance behavior as an attempt to reduce pain is common amongst persons with degenerative lumbar disorders and low back pain [12, 55]. The results on the physical measures can therefore be influenced both by a real change in physical capacity and by behavioral components as for example fear avoidance. Studies indicates that interventions for low back pain that addressed fear-avoidance beliefs are more effective than interventions based on only biomedical concepts [55] and treatment including self-management and psychological approaches are recommended [56]. Nielsen et al. [50] reported that pre-surgery self-rated physical function enhanced in patients who executed a daily home exercise-program for two months. This compared to the supervised training in the physiotherapy clinic performed in the present study in order to enhance self-efficacy, reduce avoidance behavior and ensure progression and dose of the training. Considering these results, it is of interest to further examine what specific components and mechanisms in the physical therapy lead to a successful intervention.

The significant improvements in the PT-group and the significant differences between the groups indicate that this patient group with long standing pain problems and large physical impairments tolerate and can benefit from a structured rehabilitation period. Preoperative physical fitness, physical activity and self-rated health predicts better surgical outcome in general [21, 22, 57] and after spinal surgery [47]. Therefore, preventing physical deterioration and making the patient better physically prepared for the surgical procedure and the following rehabilitation while waiting for surgery may lead to better post-surgery outcomes. Previous studies [50, 58] have not shown effects of pre-surgery intervention on long term postsurgical pain and physical function after lumbar surgery. Although they differ in intervention and study population compared to each other and to the PREPARE study [24]. Like previous literature [36–38] no side-effects from the physiotherapy intervention where reported. Lindbäck et al. [25] reports that only 58% of the patients in the PREPARE study had ≥1visit to a physiotherapist or other caregivers in the last 12 months before assessment for spinal surgery. It is important to follow the guideline recommendation to exhaust non-surgical interventions before decision on surgery. It is also obvious that the knowledge concerning the role of physical function still is scarce and further development of pre-surgery interventions is needed.

The study results should be interpreted with consideration to the methodological strength and limitations of the study. The physiotherapy intervention was planned to meet the different needs in heterogenous population and therefore the tailored physiotherapeutic intervention by the use of TBC and tailored exercises can be considered to be a strength. It is important in studies of physiotherapy interventions that the interventions are performed according to the instructions. The supervised training, check-list and follow-up meetings aimed to ensure dosage and progression of the intervention. On the other hand, treatment was given at eleven physiotherapy clinics giving a risk for disparity.

The gait test used in the study fulfills the demands of using a valid clinical measure, reflecting a part of patients’ walking capacity. It is associated with other physical functions for example aerobic capacity, muscle strength, postural control, and individuals’ general health status [29, 30]. It could be of interest to add further measures of walking distance as suggested by Tomkins-Lane et al. [59] and Ammendolia et al. [26]. Although, the results from objective and self-reported measures in present study are consistent and support the conclusion of effects on walking.

The inclusion of patients was dependent on the surgeons decision and they may not be representative for the entire spine surgeon community. Consensus is lacking regarding indications or guidelines for surgical treatment for this patient group [51, 60].

There was a drop-out of 19% in the PT-group and 7% in the waiting-list group.

The reason for drop-out were unknown. Although there were no specific patient groups amongst the drop-out regarding gender or diagnosis.

Previous results from the PREPARE study [25] showed a significant higher self-reported physical activity level pre- and post-surgery in the PT-group. The current study showed that pre-surgical physical activity level, Quadriceps strength, walking speed and walking ability together have a moderate multivariate association with physical activity level one year after surgery. The only independent variable that had a significant association was pre-surgery level of physical activity. This highlights the importance of assessing patients´ physical activity level and to focus on increasing or maintaining physical activity level as an important part in a pre-surgical intervention. It is of interest to further investigate the significance of pre-surgery physical measures and physical activity levels for the longitudinal outcome regarding physical activity level post-surgery.

In conclusion, the results of this secondary analysis on physical outcomes of the PREPARE study [24] showed that the multidimensional pre-surgery intervention had positive effects on walking ability and Quadriceps strength. Even with fairly small effect sizes, these effects might be of importance in a pre-surgery phase with risk of deterioration of physical capacity. The results confirm the previously reported outcomes on self-reported measures [25].

There is still however a need for further studies to analyse the most important components in a pre-surgery program and to study dose-effect relationships in exercises.

## Conclusion

Pre-surgery physiotherapy increased walking ability and lower extremity strength in patients with degenerative lumbar spine disorders compared to waiting-list controls. A clear treatment dose-response response relationship was not found. These results implicate that pre-surgery physiotherapy can influence functional capacity before surgical treatment and has moderate associations with maintained postoperative physical activity levels mostly explained by physical activity level pre-surgery.

## Data Availability

The dataset used and/or analysed during the current study are available from the corresponding author on reasonable request.

## Abbreviations

10MWT: Ten-meters walk test
CI: Confidence interval
DDD: Degenerative disc disease
LSS: Lumbar spinal stenosis
MRI: Magnetic resonance imaging
ODI: Oswestry disability index
PT: Physiotherapist
SD: Standard deviation
TBC: Treatment-based classification
